# Hyperspectral imaging in living and deceased donor kidney transplantation

**DOI:** 10.1186/s12880-025-01576-6

**Published:** 2025-01-31

**Authors:** Rasmus Wrigge, Robert Sucher, Fabian Haak, Hans-Jonas Meyer, Julia Unruh, Hans-Michael Hau, Matthias Mehdorn, Hans-Michael Tautenhahn, Daniel Seehofer, Uwe Scheuermann

**Affiliations:** 1 Department of Visceral, Transplantation, Vascular and Thoracic Surgery, University Hospital of Leipzig, Liebigstrasse 20, Leipzig, 04103 Germany; 2 Department of General-, Visceral- and Transplant Surgery, Medical University of Graz, Graz, Austria; 3 Department of Diagnostic and Interventional Radiology, University Hospital of Leipzig, Leipzig, Germany

**Keywords:** Hyperspectral imaging, Living donor, Deceased donor, Kidney transplantation, Delayed graft function, Outcome

## Abstract

**Objective and background:**

Hyperspectral imaging (HSI) is an innovative, noninvasive technique that assesses tissue and organ perfusion and oxygenation. This study aimed to evaluate HSI as a predictive tool for early postoperative graft function and long-term outcomes in living donor (LD) and deceased donor (DD) kidney transplantation (KT).

**Patients and methods:**

HSI of kidney allograft parenchyma from 19 LD and 51 DD kidneys was obtained intraoperatively 15 minutes after reperfusion. Using the dedicated HSI TIVITA Tissue System, indices of tissue oxygenation (StO_2_), perfusion (near-infrared [NIR]), organ hemoglobin (OHI), and tissue water (TWI) were calculated and then analyzed retrospectively.

**Results:**

LD kidneys had superior intraoperative HSI values of StO_2_ (0.78 ± 0.13 versus 0.63 ± 0.24; *P* = 0.001) and NIR (0.67 ± 0.10 versus 0.56 ± 0.27; *P* = 0.016) compared to DD kidneys. Delayed graft function (DGF) was observed in 18 cases (26%), in which intraoperative HSI showed significantly lower values of StO_2_ (0.78 ± 0.07 versus 0.35 ± 0.21; *P* < 0.001) and NIR (0.67 ± 0.11 versus 0.34 ± 0.32; *P* < 0.001). Receiver operating characteristic curve analysis demonstrated an excellent predictive value of HSI for the development of DGF, with an area under the curve of 0.967 for StO_2_ and 0.801 for NIR. Kidney grafts with low StO_2_ values (cut-off point 0.6) showed reduced renal function with a low glomerular filtration rate and elevated urea levels in the first two weeks after KT. Three years after KT, graft survival was also inferior in the group with initially low StO_2_ values.

**Conclusion:**

HSI is a useful tool for predicting DGF in living and deceased KT and may assist in estimating short-term allograft function. However, further studies with expanded cohorts are needed to evaluate the association between HSI and long-term graft outcomes.

**Supplementary Information:**

The online version contains supplementary material available at 10.1186/s12880-025-01576-6.

## Introduction

Despite improvements in organ preservation, surgical techniques and immunosuppressive therapy, many grafts following kidney transplantation (KT) still show impaired early posttransplant organ function and poor long-term graft survival. Although many donor and recipient risk factors have been identified to improve organ allocation and estimate postoperative graft function [1, 2], there are still unpredictable individual outcomes. Thus, some grafts exhibit delayed graft function (DGF), often associated with poor allograft survival [3, 4].

Hyperspectral imaging (HSI), a new, non-invasive measurement technique that allows quantitative analysis of several tissue parameters, such as tissue perfusion, has shown encouraging findings in multiple surgical applications [5–8]. By providing color images of the surgical field representing oxygenation saturation (StO_2_), tissue perfusion (near-infrared [NIR] perfusion index), organ hemoglobin index (OHI), and tissue water index (TWI), the quality of allograft reperfusion can be visualized using HSI [9]. Two previous studies revealed great potential for predicting DGF in deceased donor (DD) KT [10, 11]. However, HSI has never been tested in living donor (LD) KT, and no studies have reviewed its predictive value for long-term allograft outcomes.

Therefore, our study aimed to demonstrate and compare the short- and long-term outcomes of patients receiving DD and LD KT based on intraoperative HSI.

## Patients and methods

### Data collection and study population

Medical data from all adult patients (≥ 18 years of age) who underwent initial LD or DD KT at the University Hospital of Leipzig between January 2019 and December 2020 were retrospectively analyzed. Exclusion criteria were a multiple organ transplant or a previous transplant. Follow-up data were collected until March 2024. The data source was a prospectively collected structured query language database. The study was approved by the institutional review board of the Medical University of Leipzig (AZ 111-16-14032016).

Patient characteristics evaluated in the study population included donor and recipient age, gender, body mass index (BMI, weight in kg/ height in m^2^), donor cause of death, recipient cause of end-stage renal disease (ESRD), and dialysis duration.

The estimated glomerular filtration rate (GFR) was calculated with the Chronic Kidney Disease Epidemiology Collaboration (CKD-EPI) equation (mL/ min/ 1.73 m^2^ of standard body surface area) using serum creatinine levels [12].

Furthermore, DDs were categorized into standard criteria donors (SCD) and expanded criteria donors (ECD) based on Rao et al.’s definition [13]. All donors under the age of 50 years were defined as SCDs, and all donors older than 60 years were described as ECDs, independent of the cause of death. Donors between 50 and 59 years of age with at least two of the following criteria: history of hypertension, cerebrovascular death, and serum creatinine levels over 1.5 mg/dL were categorized as ECDs, all others were classified as SCDs [13].

The investigated perioperative and posttransplant data included surgery time, cold ischemia time (CIT), and warm ischemia time (WIT) of the kidneys. Notably, CIT is defined as the duration from the beginning of organ cold perfusion until the organ is removed from ice, while WIT is the time from cross-clamping until cold perfusion plus the anastomosis time (organ out of ice until reperfusion). During anastomosis time, cold lap pads irrigated with cold saline were utilized to prevent the warming of the organs.

Organ procurement, transplantation, induction therapy and initial immunosuppression were performed as described previously [2]. Adequate intravenous fluid supply ensures sufficient mean arterial pressure (80 mmHg) during organ reperfusion.

### Outcome measures

The outcome data included DGF, initial non-function (INF), biopsy-proven or clinically suspected and accordingly treated acute rejection in the first year after KT, date and cause of graft failure, and patient death. INF was defined as dialysis dependence or creatinine clearance ≤ 20 mL/ min at three months post-transplant. DGF was defined as the need for dialysis in the first week following transplantation [14]. Graft failure was described as a return to dialysis or pre-emptive transplant. Post-operative deaths included all deaths occurring within 30 days after surgery.

### Intraoperative kidney reperfusion assessment with HSI

Non-invasive hyperspectral images acquisition was performed intraoperatively using the TIVITA® Tissue HSI system (Diaspective Vision, Am Salzhaff, Germany), as described earlier by our study group [10].

The HSI method is based on the physical principles of optical remission spectroscopy. After being irradiated with white light in the visible and NIR range (400–100 nm), inhomogeneities in the tissue structure result in contrasting light scattering, while various tissue compounds can alter light absorption. Therefore, various tissue properties result in the remission of light with a range of wavelengths detectable by the HSI camera system.

As hemoglobin and water also impact incident light, the technique allows the determination of the perfusion state of the examined kidneys, calculating indices for the organ’s oxygenation, perfusion, hemoglobin, and water content [15].

After darkening the surrounding light sources in the operating room to ensure undisturbed image acquisition and data generation, pictures with a high spectral resolution (5 nm) in the visible and NIR range (500–1,000 nm) where obtained 15 minutes after reperfusion using the push broom HSI system.

The camera was located 30 cm from the transplanted organ with a moving arm to acquire the image using a 25-mm focal lens. Exact positioning was ensured by the device’s integrated electro-optical distance measurement system, resulting in a field of view (FOV) of 6.4 × 4.8 cm² and creating pictures with 640 × 480 (x- and y-axes) effective pixels (spatial resolution of 0.1 mm/pixel).

**Fig. 1:**
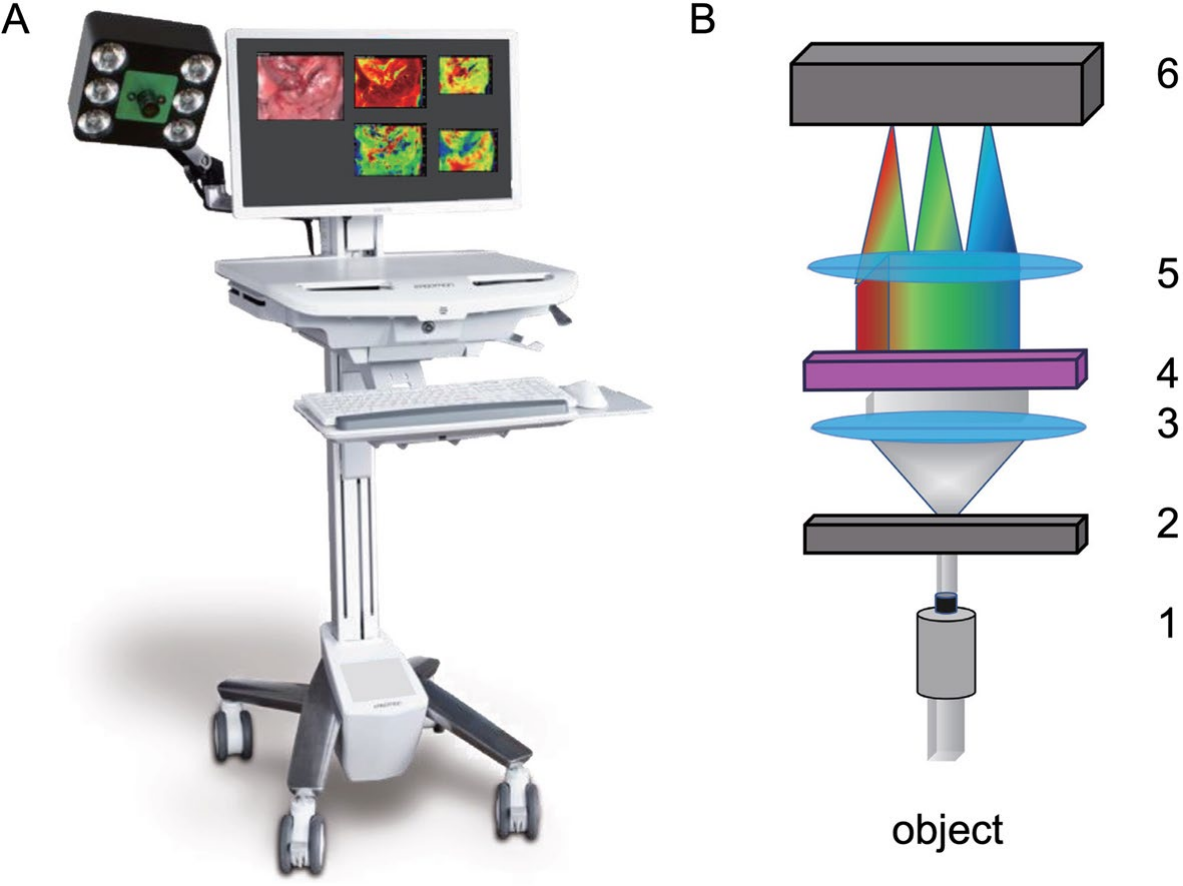
TIVITA® system for medical hyperspectral imaging (HSI). **A** TIVITA® camera system is installed on a mobile cart. The camera is attached to a holding arm and has an integrated light source. The spectrograph inside the camera is moved over the image plane for HSI measurement. The acquired HSI-Data can be displayed as false-color images on the monitor intraoperatively. **B** Reflected light from the measured object is captured by the objective lens (1) to focus it on the entrance slit (2) of the spectrograph. Special lenses (3 and 5) and a transmission grating (4) split the light, depending on the wavelength, into several beams. For digital processing, the beams are captured by a monochromatic image sensor (6). TIVITA® Tissue HSI system (Diaspective Vision, Am Salzhaff, Germany)

The acquired information is transformed into a hyperspectral data cube using the HSI camera system software, as described earlier (Fig. 1) [10, 16].

The software provides an RGB image and four color-coded images of the inspected kidney less than eight seconds after the acquisition, representing StO_2_, tissue perfusion (NIR), OHI, and TWI. The percentage of StO_2_ (0–100%) is presented in decimal fractions (0–1). While microcirculatory tissue oxygenation in superficial layers (up to 1 mm penetration depth) is displayed by StO_2_, the NIR perfusion index depicts renal tissue layers in 4–6mm penetration depth. The distribution of hemoglobin and water in the FOV is represented by the indices OHI and TWI [9, 15–17].

**Fig. 2:**
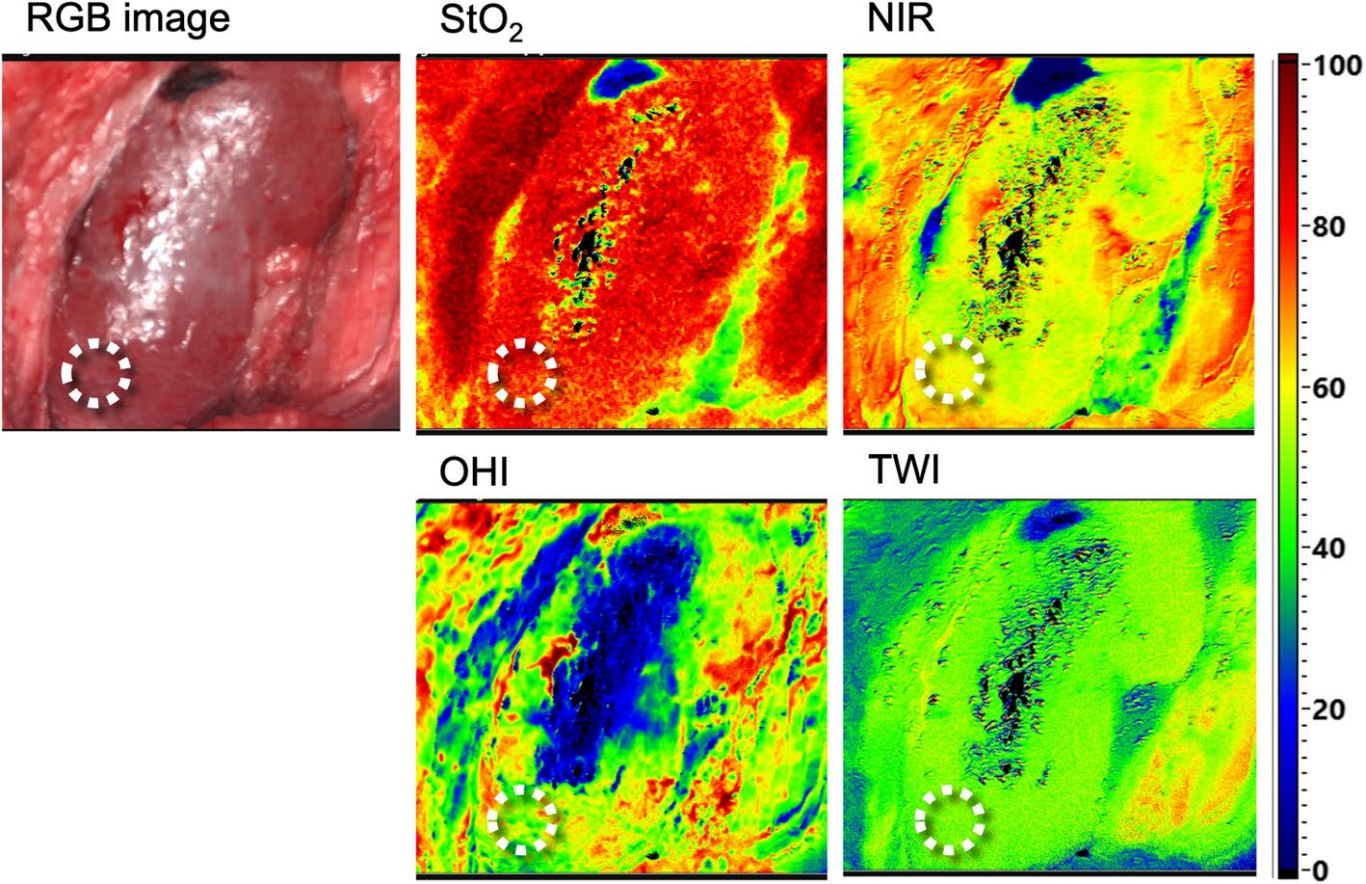
Representative intraoperative HSI images of a LD-KT allograft 15 minutes after reperfusion. RGB image and false color hyperspectral images for oxygenation (StO_2_), tissue perfusion (NIR), hemoglobin (OHI) and tissue water (TWI). The ROI (white circle) is marked within the parenchyma of kidney allograft. HSI, Hyperspectral imaging; KT, kidney transplant; LD, living donor; NIR, near-infrared perfusion index; OHI, organ hemoglobin index; RGB, red green blue; ROI, region of interest; StO2, oxygen saturation; TWI, tissue water index

After prospective collection and intraoperative review of the images generated by the TIVITA® software, they were analyzed by retrospectively inserting round markers to identify our region of interest (ROI). Areas of the kidney surface without adherent perirenal fat or light reflection artefacts were selected as ROI. For each parameter, the index average inside the ROI was calculated (Fig. 2).

### Statistical analysis

Continuous variables were compared using Welch’s t-test, while categorical variables were compared using χ² test or Fisher exact test. Multivariate Cox proportional hazard was used to evaluate independent predictors of kidney graft failure. Receiver operating characteristic (ROC) curves were used to assess the prognostic values of the HSI parameters in predicting DGF. In addition, the Youden index was employed to calculate the optimal cut-point value [18]. Diagnostic accuracy was measured by the area under the ROC curve (AUC). Higher AUC values indicate greater accuracy, whereas an AUC of 1.0 represents perfect sensitivity and specificity; an AUC of 0.5 indicates an essentially worthless test. The parametric bootstrap was used to construct confidence intervals. Survival rates were calculated using Kaplan–Meier analysis, and the log-rank test was applied to test statistical significance. Graft survival was calculated as the time from initial transplant to graft failure or patient death, additionally analyzing graft survival censoring for death with a functioning graft (death-censored graft survival). Patient survival was defined as the time from transplant to patient death, with patients still alive at the time of analysis censored. If a recipient was alive or lost to follow-up at the time of the last contact, the survival time was censored at the time of the last contact. SPSS 29.0 (SPSS Inc., Chicago, Illinois, USA) and Graph Pad Prism 10 (San Diego, CA, USA) were used for the statistical analysis. A *P*-value < 0.05 was defined as statistically significant. Baseline data are presented as mean values and standard deviations (SD).

## Results

### Study population

A total of 70 patients were included in the analysis (51 deceased and 19 living KT). The patient characteristics of the study population are shown in Table 1. Most donor and recipient parameters were similar between living and deceased KT. Compared to DD-KT recipients, recipients of LD kidneys were significantly younger (56.3 ± 13.4 years versus 44.9 ± 12.5 years; *P* = 0.002) and had spent less time on dialysis before transplant (85.7 ± 44.4 months versus 32.2 ± 31.2 months; *P* < 0.001). Four KTs (21.1%) were performed preemptively in the LD group. Twenty-six donors in the DD-KT (51%) cohort were ECDs. Four blood group-incompatible transplants were performed (21.1%) in the LD-KT cohort. Furthermore, CIT was significantly prolonged in KT after deceased donation (580 ± 222 minutes versus 138 ± 42 minutes, *P* < 0.001), whereas WITs were comparable between the groups (*P* = 0.200). There were no postoperative deaths.

**Table 1:** Donor, recipient and transplant characteristics

Variables	DD (*N* = 51)	LD (*N* = 19)	*P*-value
Donor			
Age, years	54.5 ± 17.5	57.5 ± 8.5	0.338
Gender, male/ female, n (%)	26 (51.0)/ 25 (49.0)	7 (36.8)/ 12 (63.2)	0.292
BMI, kg/m^2^	26.0 ± 3.7	27.5 ± 4.5	0.209
Comorbidity, n (%)			
Hypertension	20 (39.2)	5 (26.3)	0.317
Diabetes mellitus	1 (2.0)	2 (10.5)	0.116
Recipient			
Age, years	56.3 ± 13.4	44.9 ± 12.5	0.002
Gender, male/ female, n (%)	36 (70.6)/ 15 (29.4)	13 (68.4)/ 6 (31.6)	0.860
BMI, kg/m^2^	26.4 ± 4.1	26.2 ± 4.6	0.864
ESRD GN, n (%)	18 (35.3)	8 (42.1)	0.600
Comorbidity, n (%)			
Hypertension	41 (80.4)	14 (73.7)	0.531
Diabetes mellitus	7 (13.7)	3 (15.8)	0.826
Time on dialysis, months	85.7 ± 44.4	32.3 ± 31.2	<0.001
Transplant			
CMV D+R-, n (%)	20 (39.2)	4 (21.1)	0.155
HLA mismatch ≥4, n (%)	24 (47.1)	10 (52.6)	0.678
PRA positive, n (%)	19 (37.3)	1 (5.3)	0.006
CIT, minutes	580 ± 222.0	138 ± 41.9	<0.001
WIT total, minutes	39 ± 10.9	36 ± 5.5	0.200
Surgery time, minutes	184 ± 45.8	173 ± 39.7	0.328
Outcome			
DGF, n (%)	15 (29.4)	3 (15.8)	0.360
Hospital stay, days	23 ± 15.2	27 ± 29.9	0.530
Rejection first year, n (%)	11 (21.6)	5 (26.3)	0.752

*BMI* body mass index, *CIT* cold ischemia time, *CMV D + R-* high risk cytomegalovirus status: donor+/ recipient-, *DGF* delayed graft function, *ESRD GN* end-stage renal disease glomerulonephritis, *HLA* human leukocyte antigen, *KT* kidney transplantation, *LD* living donor, *nDGF* non-delayed graft function, *PRA* panel reactive antibody, *WIT* warm ischemia time

### Living and deceased kidney transplants

**Fig. 3:**
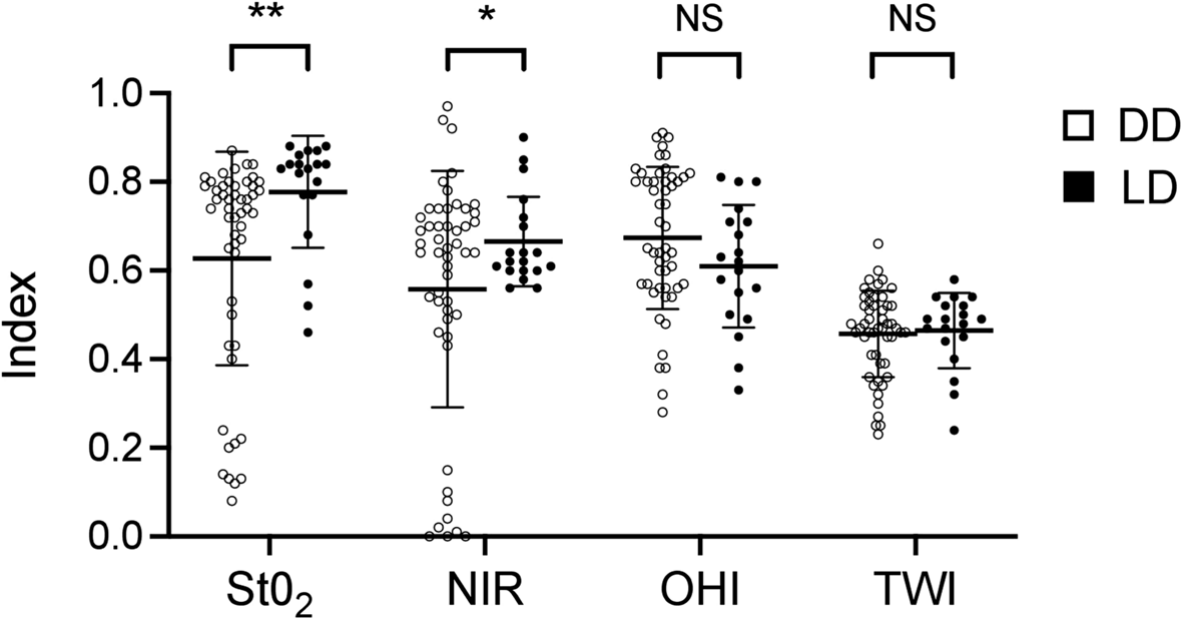
HSI-Data of the total cohort comparing DD and LD kidney grafts 15 minutes after reperfusion. DD, deceased donor; HSI, Hyperspectral imaging; LD, living donor; NIR, near-infrared perfusion index; OHI, organ hemoglobin index; StO2, oxygen saturation; TWI, tissue water index. *P < 0.05 **P < 0.01; NS, not significant

Fifteen minutes after reperfusion, the LD kidney grafts showed significantly higher values of StO_2_ (LD: 0.78 ± 0.13 versus DD: 0.63 ± 0.24; *P* = 0.001) and NIR (LD: 0.67 ± 0.10 versus DD: 0.56 ± 0.27; *P* = 0.016) than the KT after deceased donation. Furthermore, OHI and TWI showed no significant difference between living and deceased KT (Fig. 3).

### Delayed graft function

Eighteen patients (26%) showed DGF. Table 2 compares KT with and without DGF (nDGF). Donor characteristics were comparable between the groups. Transplant kidneys developing DGF were associated with a significantly prolonged CIT (*P* = 0.043), surgery time (*P* = 0.002), high recipient BMI (*P* = 0.015), and extended time on dialysis before transplantation (*P* = 0.039). There were no significant differences in the incidence rate of DGF regarding the type of organ donation (LD: 3 (16%) versus DD: 15 (29%); *P* = 0.360) or differences in DGF development when comparing ECD with SCD and LD (*P* = 0.468).

**Table 2:** Donor, recipient and transplant characteristic according to development of delayed graft function

Variables	nDGF (*N* = 52)	DGF (*N* = 18)	*P*-value
Donor			
Age, years	55.8 ± 16.4	53.9 ± 13.4	0.635
Gender, male/ female, n (%)	22 (42.3)/ 30 (57.7)	11 (61.1)/ 7 (38.9)	0.168
BMI, kg/m^2^	26.0 ± 3.9	27.5 ± 4.1	0.170
Comorbidity, n (%)			
Hypertension	17 (32.7)	8 (44.4)	0.370
Diabetes mellitus	2 (3.8)	1 (5.6)	0.758
Recipient			
Age, years	52.8 ± 14.4	54.3 ± 13.3	0.695
Gender, male/ female, n (%)	35 (67.3)/ 14 (26.9)	14 (77.8)/ 4 (22.2)	0.403
BMI, kg/m^2^	25.6 ± 3.9	28.6 ± 4.3	0.015
ESRD GN, n (%)	20 (38.5)	6 (33.3)	0.698
Comorbidity, n (%)			
Hypertension	41 (78.8)	14 (77.8)	0.924
Diabetes mellitus	5 (9.6)	5 (27.8)	0.058
Time on dialysis, months	66.0 ± 45.7	93.7 ± 46.5	0.039
Transplant			
CMV D+R-, n (%)	17 (32.7)	7 (38.9)	0.633
HLA mismatch ≥4, n (%)	24 (46.2)	10 (55.6)	0.492
PRA positive, n (%)	13 (25.0)	7 (38.9)	0.327
CIT, minutes	419 ± 245.8	593 ± 313.0	0.043
WIT total, minutes	38 ± 10.1	38 ± 9.4	0.898
Surgery time, minutes	172 ± 43.9	206 ± 35.6	0.002
Outcome			
Hospital stay, days	19 ± 12.1	39 ± 30.1	0.014
Rejection first year, n (%)	10 (19.2)	6 (33.3)	0.219

*BMI* body mass index, *CIT* cold ischemia time, *CMV D + R-* high risk cytomegalovirus status: donor+ /recipient-, *DGF* delayed graft function, *ESRD GN* end-stage renal disease glomerulonephritis, *HLA* human leukocyte antigen, *KT* kidney transplantation, *LD* living donor, *nDGF* non-delayed graft function, *PRA* panel reactive antibody, *WIT* warm ischemia time.

Regarding the three kidney grafts with DGF after LD, one graft developed necrosis on the upper kidney pole after arterial reconstruction. Another LD kidney graft developed a subcapsular hemorrhage immediately after reperfusion. In addition, the third patient who developed DGF after LD exhibited a BMI of 37 kg/m^2^ and a short renal artery and vein, thus complicating the implantation procedure and potentially being responsible for the impaired reperfusion.

After the transplant, patients developing DGF spent significantly increased time in the hospital before discharge (18.9 ± 12.1 days versus 38.6 ± 30.1 days; *P* = 0.014). Seven DD-KT recipients and one LD-KT recipient showed INF (*P* = 0.243).

**Fig. 4:**
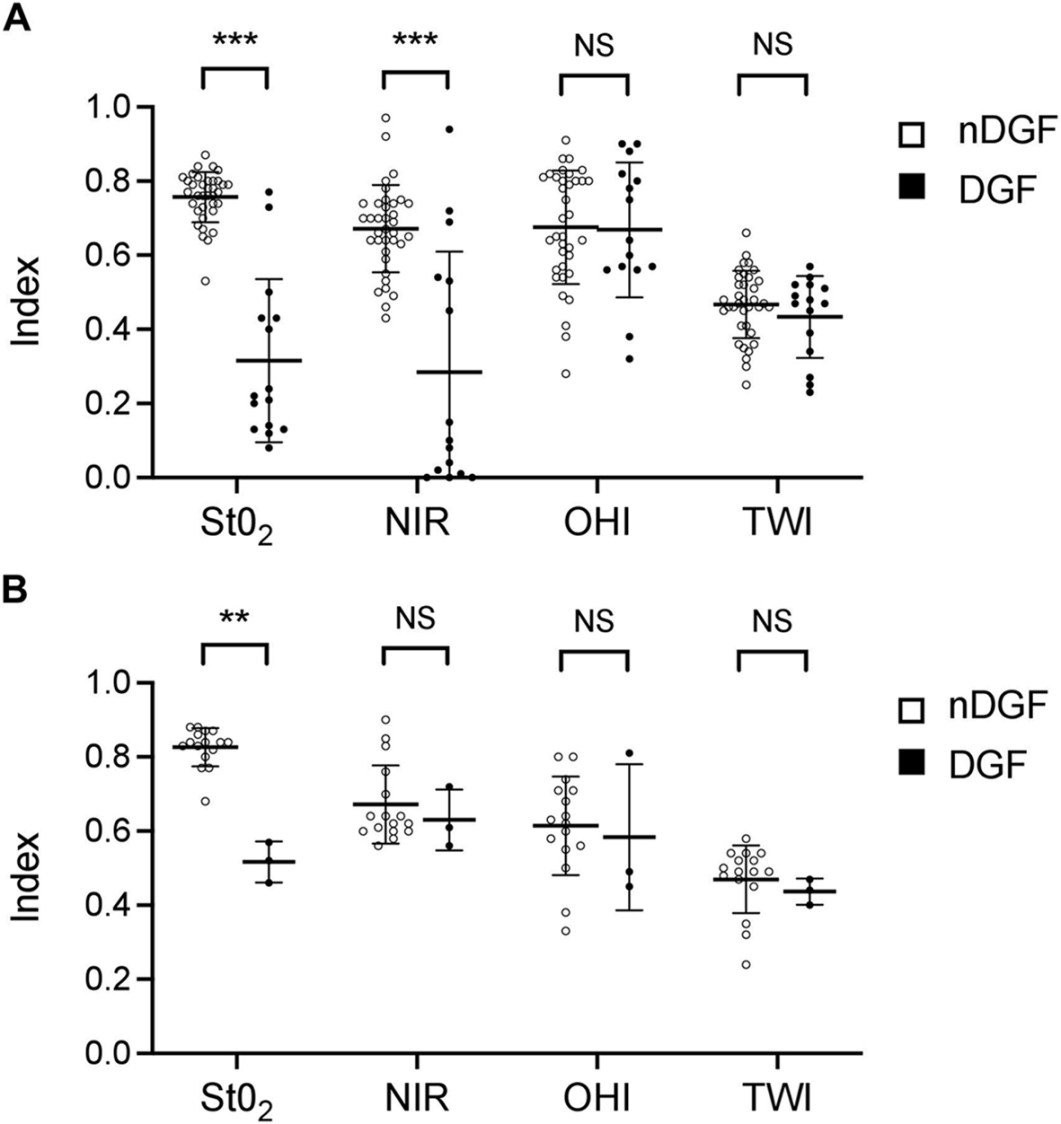
HSI-Data of the **A** deceased donor and **B** living donor kidney transplant cohort according to occurrence of DGF. DD, deceased donor; DGF, delayed graft function; HSI, Hyperspectral imaging; LD, living donor; nDGF, non-delayed graft function; NIR, near-infrared perfusion index; OHI, organ hemoglobin index; StO_2_, oxygen saturation; TWI, tissue water index. **P < 0.01 ***P < 0.001; NS, not significant

In the overall study population, intraoperative HSI showed significantly reduced values of StO_2_ (0.78 ± 0.07 versus 0.35 ± 0.21; *P* < 0.001) and NIR (0.67 ± 0.11 versus 0.34 ± 0.32; *P* < 0.001) in grafts developing DGF. No significant differences were observed between the groups regarding the other HSI parameters (OHI and TWI). In the subgroup analysis, the same was observed in the DD-KT cohort (StO_2_: 0.76 ± 0.07 versus 0.32 ± 0.22; *P* < 0.001; NIR: 0.67 ± 0.12 versus 0.29 ± 0.32; *P* < 0.001) (Fig. 4A), whereas LD kidneys developing DGF only showed reduced values for StO_2_ (0.83 ± 0.05 versus 0.52 ± 0.06; *P* = 0.006) (Fig. 4B).

**Fig. 5:**
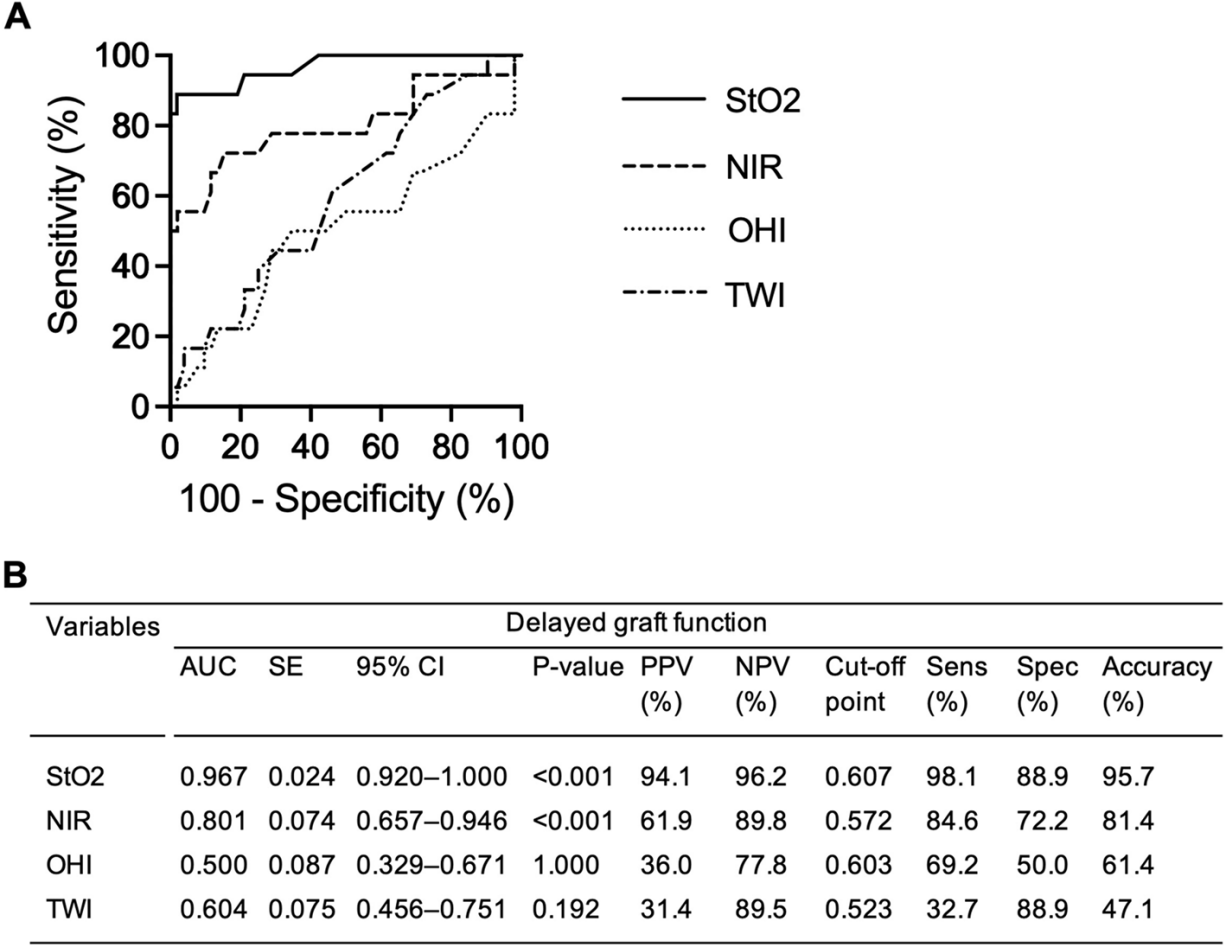
**A** Receiver operating characteristic (ROC) curves and **B** analysis of HSI-data for development of delayed graft function. AUC, area under the curve; HSI, Hyperspectral imaging; NIR, near-infrared perfusion index; NPV, negative predictive value; PPV, positive predictive value; OHI, organ hemoglobin index; SE, standard error; Sens, sensitivity; Spec, specificity; StO_2_, oxygen saturation; TWI, tissue water index.; 95% CI, 95% confidence interval

The ROC curves and analysis of test accuracy are presented in Fig. 5. Notably, AUCs were 0.967 for StO_2_ and 0.801 for NIR, with sensitivity and specificity of 98.1% and 88.9% for StO_2_ and 84.6% and 72.2% for NIR, respectively. Other parameters had limited predictive values. Bootstrapping analysis of HSI Data are presented in Suppl. Table 1.

### Renal function

LD und DD-KT cohorts showed similar values for urea and GFR on postoperative day 6 (Urea: LD: 14.9 ± 11.4 versus DD: 16.6 ± 8.7; *P* = 0.571; GFR: LD: 38.6 ± 24.9 versus DD: 27.8 ± 18.4; *P* = 0.096) and postoperative day 14 (Urea: LD: 11.2 ± 6.1 versus DD: 12.9 ± 8.9; *P* = 0.371; GFR: LD: 44.0 ± 22.9 versus DD: 36.5 ± 19.0; *P* = 0.212).

**Fig. 6:**
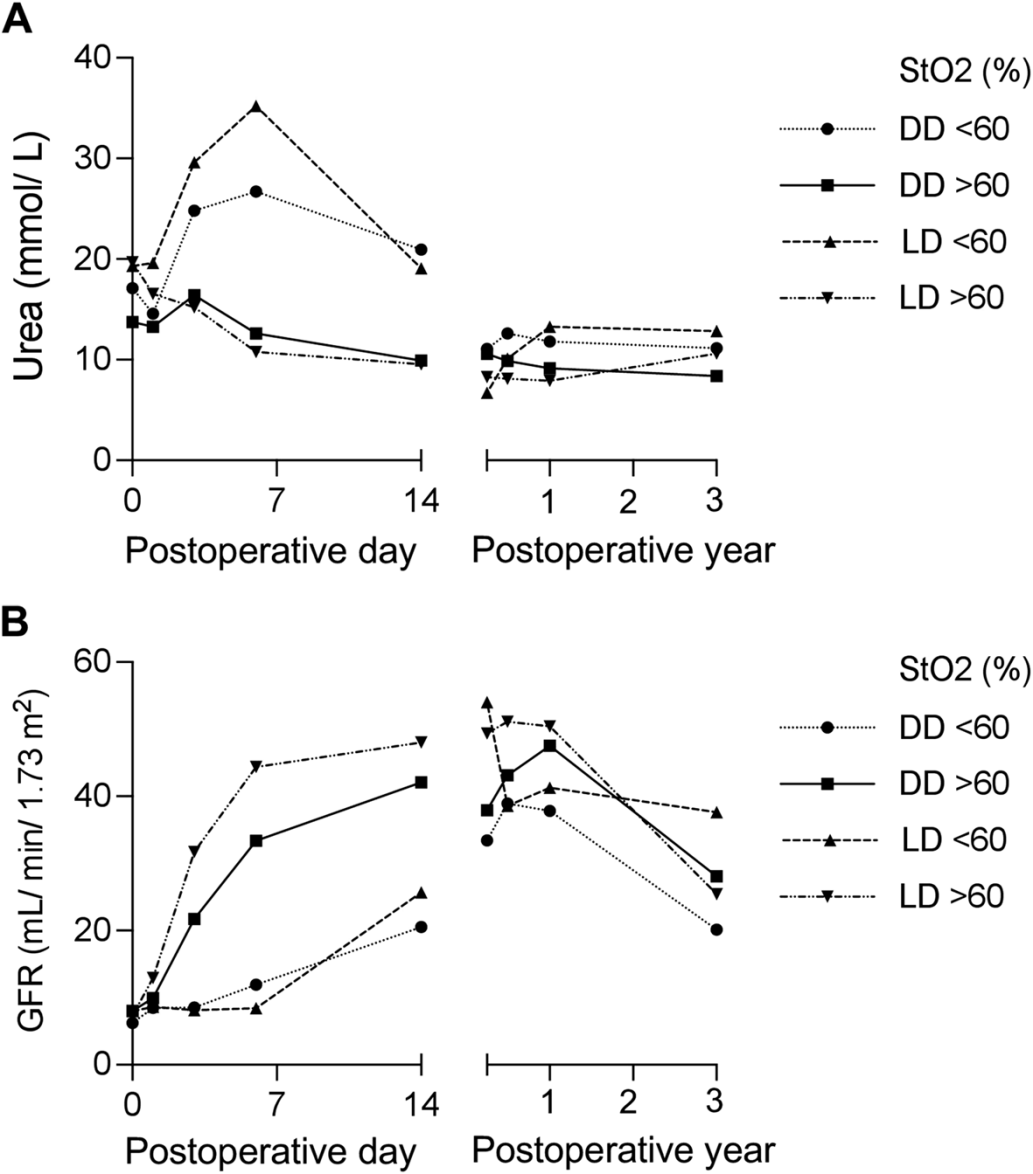
Mean post-operative **A** urea levels and **B** glomerular filtration rates according to transplantation mode and intraoperative StO_2_. GFR, glomerular filtration rate; LD, living donor; DD, deceased donor; StO2, oxygen saturation

For long-term outcome evaluation, we divided the cohort into two groups above and below the optimal cut-point value of StO_2_ (StO_2_ low and high) for the development of DGF (optimal cut-point value: StO_2_ = 60.7%). Figure 6 shows the GFR and urea values among the groups within the first three years after KT. Recipients with low StO2-values in the LD and DD-KT cohorts had reduced values in the first two weeks after KT. No long-term differences were observed between the groups.

Due to limited sample size, an analysis of the correlation between NIR cut-off value and DD and LD renal function was not possible.

### Long-term survival

The one- and three-year cumulative graft survival rates were 88.6% and 81.4%, respectively. According to initial StO_2_, one- and three-year graft survival rates were 76.5% and 64.7% for the low (*N* = 17) StO_2_ group and 92.5% and 86.8% for the high (*N* = 53) StO_2_ group (*P* = 0.037), respectively.

Cumulative death-censored graft survival rates were 95.7% and 91.4% after one and three years, respectively. Based on initial StO_2_, death-censored one- and three-year graft survival rates were 82.4% and 76.5% for the low (*N* = 17) StO_2_ group and 100% and 96.2% for the high (*N* = 53) StO_2_ group (*P* = 0.009), respectively.

Reasons for graft loss included rejection (*N* = 2), fulminant graft infection (*N* = 1), venous thrombosis of the transplant kidney (*N* = 1) and septic shock (*N* = 1). In one case, the cause of graft loss remained unclear. All cases of graft loss occurred in kidney grafts after deceased donation.

The overall one- and three-year patient survival rates were 90.0% and 85.7%, respectively. The patient survival rates one and three years after KT were 82.4% and 70.6% in the group with low StO_2_ and 92.5% and 90.6% in the group with high StO_2_ (*P* = 0.046), respectively.

The causes of death included septic shock (*N* = 5), stroke (*N* = 2), cardiac arrest (*N* = 1), and suicide (*N* = 1). In one case, the cause of death was not ascertainable.

Assessment of long-term survival according to NIR, OHI and TWI cut-point values did not result in significant differences (*P* > 0.05 each).

## Discussion

### HSI in living kidney transplantation

This is the first study to apply HSI in LD-KT and to evaluate the long-term outcomes of KT recipients based on HSI.

In our study, LD kidney grafts showed significantly higher values of StO_2_ and NIR after organ reperfusion than KT after deceased donation. The reasons for this observation are likely multifactorial. Generally, superior organ quality and shorter CITs contribute to the better outcome seen in living KT, as both factors could prevent a severe ischemia-reperfusion injury (IRI) and consecutively lead to improved microtissue perfusion and oxygenation. Moreover, as seen in our study, surgical complications, such as vascular occlusion and subcapsular hemorrhage after reperfusion or a longer WIT in cases of complicated implantation, can contribute to poor intraoperative HSI values and development of DGF.

### Prediction of postoperative renal function

Pretransplant, the donor’s and recipient’s characteristics can help estimate future allograft function. However, they do not consider any impairment of the kidney during the transplantation process and thus are limited in predicting allograft outcomes [19]. The HSI measurement after organ reperfusion can consider these risk factors and therefore provide crucial information about future organ function. Consequently, HSI could be used as an alternative or additional tool to established methods, such as protocol biopsies, during graft implantation. Especially as biopsies, unlike HSI measurement, are invasive and studies have shown that it does not benefit patients with a low risk of rejection [20, 21].

Notably, DGF is a prevalent complication in kidney allografts, associated with poor graft performance and suboptimal long-term outcomes for recipients [3, 22]. Moreover, DGF leads to extended hospitalization, prolonged stays in intensive care units, and increased costs [23]. Previous studies have reported a strong relationship between intraoperative HSI and the development of DGF in DD-KT [10, 11]. We confirmed these findings in LD-KT.

A major factor contributing to DGF development is IRI, as restored blood flow damages the kidney parenchyma after prolonged ischemia [14, 24]. In organs with impaired graft function, it is challenging to distinguish between DGF resulting from IRI and organs requiring immediate therapy, such as grafts with acute rejection. Our study demonstrated reduced values for StO_2_ and NIR in transplant kidneys developing DGF, possibly suggesting impaired microcirculatory tissue perfusion in the affected kidneys. 

After KT, allograft function is estimated using recipient serum creatinine levels or GFR. Nevertheless, this biomarker tends to increase only in the late stages of injury, limiting its utility in detecting early graft dysfunction [25]. Promising new noninvasive biomarkers, such as neutrophil gelatinase-associated lipocalin, measured in urine samples, can indicate graft damage in kidneys with IRI. However, they are not yet used in daily clinical routines because their analytic process is too complex and protracted [26]. Therefore, HSI, especially as a noninvasive method, is beneficial for additional organ assessment. The ability to detect early graft dysfunction would allow further diagnostic steps and close monitoring of the affected kidneys.

### Graft and patient survival

For the first time, patients who underwent KT with intraoperative HSI measurement were surveilled for more than 12 months [10, 11]. Patients with reduced intraoperative StO_2_ values showed inferior graft and patient survival compared to those with higher StO_2_ values. However, our study cohort is too small to draw definitive conclusions about the predictive ability of HSI. More studies with expanded patient numbers are needed to evaluate this further.

Other diagnostic methods, such as the resistive index measured by Doppler sonography, have also been tested in the KT setting; however, they have shown controversial results regarding their ability to predict short- and long-term outcomes [27–30]. Indocyanine green (ICG) angiography may also represent a diagnostic tool for displaying microperfusion and predicting future graft outcomes [31]. However, recent studies have found no significant association between ICG and graft survival after one year [32].

### Future perspective

Currently, obtaining real-time data is not feasible, as processing HSI images is a prerequisite. Real-time HSI could allow an immediate intraoperative evaluation of the kidney parenchyma. In combination with artificial intelligence [33, 34], further applications of HSI are conceivable, such as organ quality assessment during ex vivo machine perfusion before transplant. The pretransplant evaluation of ECD kidneys would be particularly relevant in this context. Finally, HSI could support the transplant surgeon in challenging graft implantations (for instance, in organs needing difficult vascular reconstruction) or during organ reperfusion to adjust the inflow pressure.

### Limitations

The major limitations of our study are the small sample size and its single-center character. Therefore, survival analysis has low statistical power, and multivariate Cox regression was not suitable. A further limitation was the measurement of a single time point, which precluded the possibility of conducting a time dependent HSI analysis. Regarding the technique, HSI lacks the capability for continuous postoperative organ monitoring, as a close, direct view of the organ is necessary. Furthermore, the low penetration depth only represents superficial tissue layers, providing no information about the entire organ. Therefore, calibration with the results of another diagnostic tool, such as Doppler ultrasound, should be performed to validate the HSI data.

## Conclusion

HSI is an efficient, non-invasive tool for quantitative assessment of micro-perfusion and oxygenation, allowing objective evaluation of graft viability after reperfusion. However, further studies are necessary to validate HSI-data and its association with long-term graft survival, especially in LD-KT.

## Supplementary Information

Supplementary Material 1: Supplemental Table 1. Bootstrap analysis of HSI-data for development of delayed graft function.

## Data Availability

Our database contains highly sensible data which may provide insight in clinical and personnel information about our patients and lead to identification of these patients. Therefore, according to organizational restrictions and regulations these data cannot be made publicly available. However, the datasets used and/or analyzed during the current study are available from the corresponding author on reasonable request.

## Abbreviations

AUC: Area under the curve
BMI: Body mass index
CI: Confidence interval
CIT: Cold ischemia time
CVA: Cerebrovascular accident
DD: Deceased donor
DGF: Delayed graft function
ECD: Expanded criteria donor
ESRD: End-stage renal disease
FOV: Field of view
GFR: Glomerular filtration rate
GN: Glomerulonephritis
HSI: Hyperspectral imaging
ICU: Intensive care unit
INF: Initial non-function
IRI: Ischemia reperfusion injury
KT: Kidney transplantation
LD: Living donor
nDGF: Non-delayed graft function
NIR: Near-infrared perfusion index
OHI: Organ hemoglobin index
RGB: Red, green, blue
RI: Resistive index
ROC: Receiver operating characteristic
ROI: Region of interest
SCD: Standard Criteria donor
SD: Standard deviation
StO_2_: Tissue oxygenation
TWI: Tissue water index
WIT: Warm ischemia time
